# Predicting progression of white matter hyperintensity using coronary artery calcium score based on coronary CT angiography—feasibility and accuracy

**DOI:** 10.3389/fnagi.2023.1256228

**Published:** 2023-11-10

**Authors:** Hui Jin, Jie Hou, Xue Qin, Xingyue Du, Guangying Zheng, Yu Meng, Zhenyu Shu, Yuguo Wei, Xiangyang Gong

**Affiliations:** ^1^Department of Radiology, Center for Rehabilitation Medicine, Zhejiang Provincial People’s Hospital, Affiliated People’s Hospital, Hangzhou Medical College, Hangzhou, Zhejiang, China; ^2^Bengbu Medical College, Bengbu, China; ^3^Advanced Analytics, Global Medical Service, GE Healthcare, Hangzhou, China

**Keywords:** white matter hyperintensity, coronary artery calcium, coronary CT angiography, risk factors, retrospective studies

## Abstract

**Objective:**

Coronary artery disease (CAD) usually coexists with subclinical cerebrovascular diseases given the systematic nature of atherosclerosis. In this study, our objective was to predict the progression of white matter hyperintensity (WMH) and find its risk factors in CAD patients using the coronary artery calcium (CAC) score. We also investigated the relationship between the CAC score and the WMH volume in different brain regions.

**Methods:**

We evaluated 137 CAD patients with WMH who underwent coronary computed tomography angiography (CCTA) and two magnetic resonance imaging (MRI) scans from March 2018 to February 2023. Patients were categorized into progressive (*n* = 66) and nonprogressive groups (*n* = 71) by the change in WMH volume from the first to the second MRI. We collected demographic, clinical, and imaging data for analysis. Independent risk factors for WMH progression were identified using logistic regression. Three models predicting WMH progression were developed and assessed. Finally, patients were divided into groups based on their total CAC score (0 to <100, 100 to 400, and > 400) to compare their WMH changes in nine brain regions.

**Results:**

Alcohol abuse, maximum pericoronary fat attenuation index (pFAI), CT-fractional flow reserve (CT-FFR), and CAC risk grade independently predicted WMH progression (*p* < 0.05). The logistic regression model with all four variables performed best (training: AUC = 0.878, 95% CI: 0.790, 0.938; validation: AUC = 0.845, 95% CI: 0.734, 0.953). An increased CAC risk grade came with significantly higher WMH volume in the total brain, corpus callosum, and frontal, parietal and occipital lobes (*p* < 0.05).

**Conclusion:**

This study demonstrated the application of the CCTA-derived CAC score to predict WMH progression in elderly people (≥60 years) with CAD.

## Introduction

White matter hyperintensity (WMH) is a prevalent manifestation in cerebral small vessel disorders. The pathogenesis of WMH is interconnected with various pathological conditions that impact the small arteries, capillaries, and small veins that perfuse brain tissue, causing damage to both gray and white matter (Wardlaw et al., 2013a). This type of brain injury appears as hyperintense signal changes on T2-weighted and fluid-attenuated inversion recovery (FLAIR) sequences of magnetic resonance imaging (MRI) scans (Wardlaw et al., 2015). The prevalence of WMH increases with age, and among otherwise healthy individuals aged 80 or older, about 64–94% have WMH (Garde et al., 2000). The rate is even higher among individuals with a history of cardiovascular risks, diagnosed heart disease, or kidney impairment (Liu et al., 2018; Moroni et al., 2018). The presence and severity of WMH are associated with an increased risk of dementia, cognitive impairment and stroke, indicating that WMH may be a target for intervention in the management of small vessel disease (Debette and Markus, 2010). Therefore, it is crucial to identify the risk factors for WMH so we can intervene early to prevent or delay their onset (Ter Telgte et al., 2018; Chung et al., 2023).

Cardiovascular disease, including coronary artery disease (CAD), is the primary cause of mortality in developed nations (Sanchis-Gomar et al., 2016). It is estimated that 4 million patients die of cardiovascular causes in Europe every year (Townsend et al., 2016). CAD is a chronic and progressive disease that is usually caused by atherosclerosis. It develops as plaque accumulates in the heart, obstructing coronary arteries partially or entirely and leading to hindered blood flow to the myocardium (Libby et al., 2011). Despite the differences in clinical manifestations between heart and brain diseases, they may share similar pathophysiological mechanisms, such as atherosclerosis in coronary arteries and cerebral small vessels, because both organs rely on large surface arteries to deliver blood through a network of small penetrating vessels (Berry et al., 2019). Identifying a connection between these two diseases would be of great significance for managing and reducing the risk of brain complications (such as WMH) in CAD patients.

The coronary artery calcium (CAC) score is a non-invasive metric derived from non-contrast coronary computed tomography angiography (CCTA) images. It measures the volume of calcium in coronary arteries to assess individual plaque accumulation (Malguria et al., 2018). The CAC score, a reliable measure of risk for cardiovascular and cerebrovascular diseases, is linked not only to coronary heart disease but also to ischemic stroke and cranial artery stenosis (Hermann et al., 2013; Oh et al., 2015). The presence and larger volume of coronary artery plaque have been associated with larger WMH volume (Johansen et al., 2021). Two other CCTA-derived markers can reflect the severity of CAD (Moroni et al., 2018): The pericoronary fat attenuation index (pFAI) is an imaging biomarker that reflects vascular inflammation (Antonopoulos et al., 2017). It remains unaffected by both CAC and lumen stenosis, and it can provide independent information on the degree of systemic inflammation and predict adverse cardiac events (Oikonomou et al., 2018, 2019). Inflammation of brain tissue is hypothesized to lead to the destruction of microcirculation, leading to WMH (Moroni et al., 2018). Apart from the shared factor of inflammation contributing to the onset and progression of these two diseases, both the brain and heart depend on adequate blood supply to meet their intense metabolic demands. Controlling resistance in the brain’s microcirculation is crucial for ensuring sufficient local blood flow in the brain (Moroni et al., 2020). The other CCTA-derived marker, CT-derived fractional flow reserve (CT-FFR) can be obtained by using fluid dynamics technology to evaluate the degree of cardiac ischemia (Min et al., 2015). Thus, abnormal perfusion in these two organs causes similar pathological changes.

The complex and multifactorial relationship between the CAC score and WMH remains unclear. It is critical to monitor changes in WMH in patients with coronary atherosclerosis, since such changes may affect their prognosis and treatment. Our preliminary research discovered a linear correlation between the CAC score and the volume of WMH in elderly populations with high clinical risk factors, which suggested that the CAC score could serve as an imaging biomarker predicting the progression of WMH (Jin et al., 2023). Therefore, this study aimed to develop an accurate WMH progression prediction model based on the CAC score along with other critical demographic and risk factors such as the CCTA-derived markers pFAI and CT-FFR.

## Materials and methods

This study was approved by the Ethics Committee of ZJPP Hospital. Due to the retrospective application of imaging data, the need for informed consent was waived. The investigation complied with the principles set out in the Declaration of Helsinki.

### Study population

**Figure 1:**
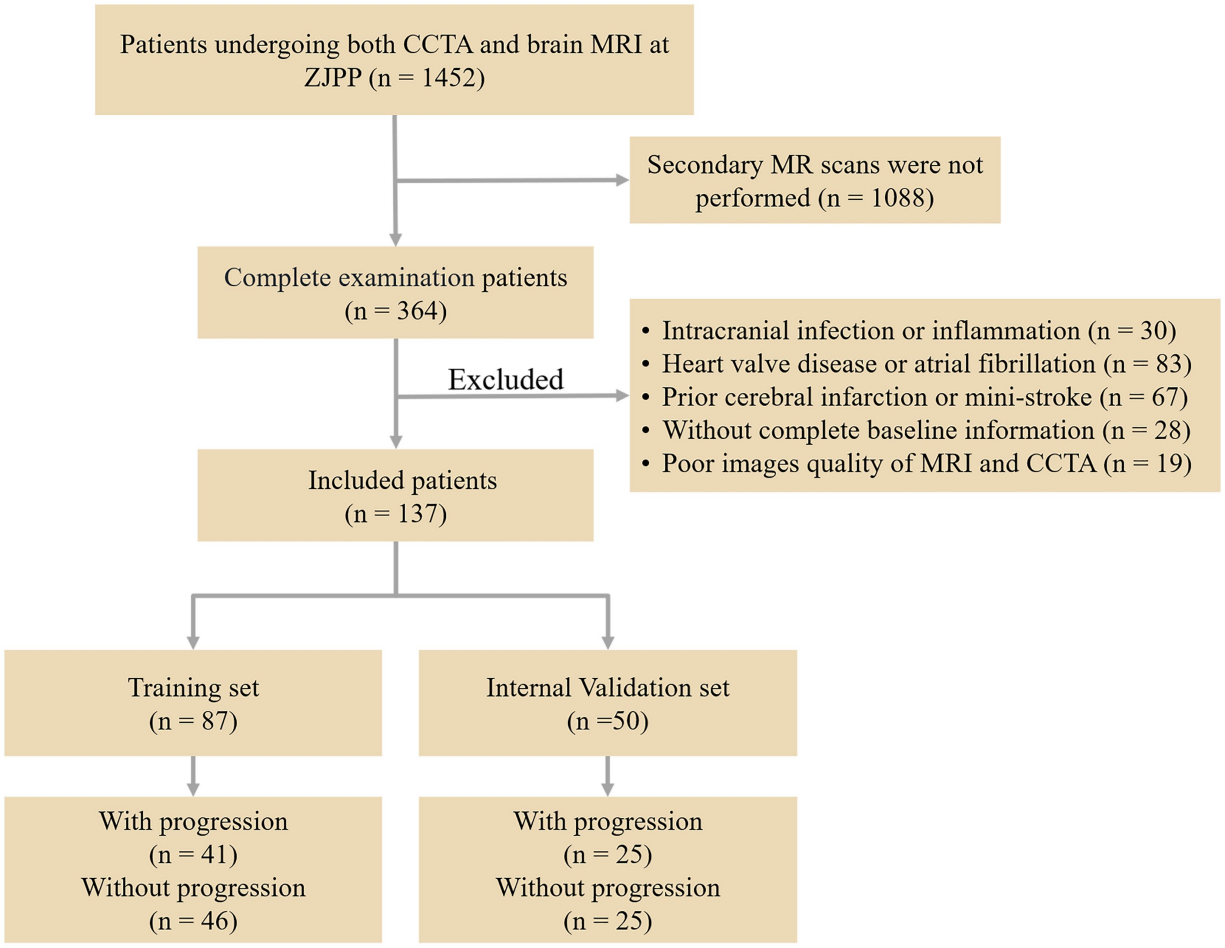
Flowchart of participant recruitment and study design.

This retrospective study gathered data from 1,452 patients who had both CCTA and brain MRI at Zhejiang Provincial People’s Hospital between March 2018 and February 2023. Of these, 364 patients had WMH and underwent a second brain MRI after a 12-month interval. Among them, 137 patients met the inclusion criteria and were allocated to the training and validation models. The inclusion criteria were as follows: (1) WMH seen on T2 FLAIR and T2 weighted MR images; (2) age 60 or above; (3) absence of stroke (not counting lacunar infarction) on the DWI sequence; (4) no neurological symptoms such as Alzheimer’s disease, multiple sclerosis, or major brain injuries; (5) no history of heart attack or coronary stenting; and (6) symptoms of typical or atypical angina persisting for at least 3 months. Exclusion criteria: (1) intracranial infection or inflammation; (2) heart valve disease or irregular heart rhythm; (3) past cerebral infarction or mini-stroke; (4) inability to provide full baseline information; and (5) poor quality of MRI and CCTA images. Figure 1 shows the flowchart of participant recruitment and study design.

### Clinical examination and definition

Demographic and clinical data of all study participants were gathered, including age, sex, body mass index (BMI), hypertension, hyperlipidemia, diabetes, smoking, and alcohol abuse. The calculation of BMI involved dividing weight (in kgs) by the square of height (in meters). The criteria for hypertension included a systolic blood pressure not less than 140 mmHg, a diastolic blood pressure at least 90 mmHg, or the regular use of hypertension medication. Hyperlipidemia was identified if the participants had total cholesterol ≥240 mg/dL, had low-density lipoprotein cholesterol ≥160 mg/dL, or were taking lipid-lowering drugs. The presence of diabetes was determined by the intake of diabetic pills or an HbA1c level of 6.5% or more (ElSayed et al., 2023). All these data were sourced from hospital information system (HIS), which is a comprehensive information management system that covers all operations and their entire process in hospital; it also includes medical records and test results.

### CCTA image acquisition

CCTA was done by using a third-generation dual-energy CT (DECT) scanner (Somatom Force; Siemens Healthcare, Erlangen, Germany). The patient was told to fast before the examination, and the patient’s heart rate, height and weight were measured. After acquisition of the localization image, the contrast agent iohexol (350 mg I/mL) was injected via the right median vein at a rate of 5.0 mL/s using a high-pressure syringe, and the contrast agent injected was 30 mL of contrast agent plus 40 mL of saline. The scan was triggered using bolus tracking, with the region of interest (ROI) placed in the ascending aorta at a threshold of 100 Hounsfield units (HU) and a 4-s delay in initiating the scan. The image quality imaging conditions were 100 kVp, 288 mAs, other scan parameters: prospective cardiac gating, 65% RR interval, pitch 3.2, 0.25 s/r, detector collimation width 192 × 0.6 mm, reconstruction layer thickness and interval 3 mm.

### Measurement of CAC score

**Figure 2:**
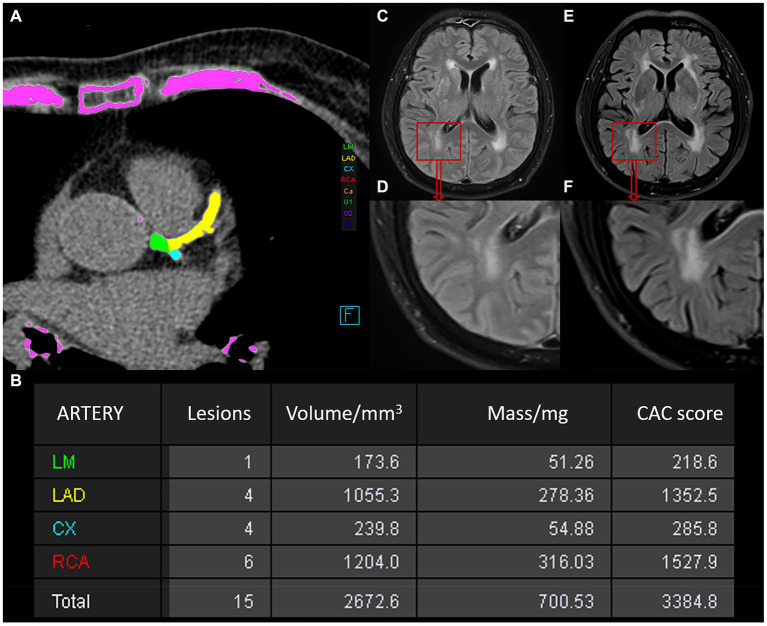
Schematic illustration of the measurements of the CAC score and WMH in a 78-year-old man. **(A)** Automatic measurement of the CAC score using Siemens software, with calcium plaques labeled in different colors. **(B)** Table of CAC score results generated by the software: the first column represents the artery, the second indicates the number of lesions, the third shows calcium volume, the fourth provides equivalent mass, and the fifth gives the score value. **(C–F)** Changes in WMH observed between two MRI examinations of the patient. **(C,D)** Show the baseline WMH on MRI. **(E/F)** Represents the follow-up MRI examination after an interval of 15 months, showing progression of WMH.

The non-contrast image sets were reconstructed (B35f HeartView medium CaScore), and CAC was identified and quantified using syngo.via calcium scoring software (Volume Wizard; Siemens). The four key coronary arteries assessed included the left main artery (LM), left anterior descending artery (LAD), right coronary artery (RCA), and left circumflex artery (LCX). We defined the total CAC score as the sum of the CAC burden of the four coronary vessels. Lesions going beyond the calcium threshold of 130 HU within a volume of 1 mm^3^, spread across at least three neighboring pixels, were detected using 3D-based tools. The areas of calcium in each slice, approximately 3 mm thickness, were bolstered by an intensity factor and added up across slices to calculate the CAC score through the Agatston method. With this method, the CAC volume was measured by multiplying the area of each lesion by a weighted attenuation score, which depends on the maximum attenuation within the lesion. Two radiologists (with 3 and 8 years of diagnostic imaging experience, respectively) reviewed and corrected the presence and amount of CAC independently, both blinded to the clinical information of all patients. The total CAC score was grouped into low-risk (score under 100), medium-risk (score between 100 and 400) and high-risk categories (score over 400). The measurement of CAC score is depicted in Figures 2A,B.

### Acquisition of CT-FFR and pFAI

We used the PHIgo workstation (Precision Health Institution, Version 1.5.1) to measure the CT-FFR value and pFAI value of the target lesion based on threshold values for a high risk of CAD (CT-FFR ≤ 0.80 and pFAI ≥ −70.1 HU) (Zhang et al., 2022). Arterial phase images of CCTA in each patient were imported in DICOM format into the CQK analysis platform of PHIgo software (Precision Health Institution, Version 1.5.1) for automated segmentation of the coronary artery (Hou et al., 2023). A simulated three-dimensional coronary artery image was obtained by automated segmentation and reconstruction of the main coronary segments of the vessel for visualization. According to the CCTA images, a three-dimensional anatomical model of the centerline and luminal contours was constructed for the coronary artery tree. Specifically, CT-FFR at the target lesion was calculated using computational fluid dynamics under the simulated maximum hyperemia condition (Khav et al., 2020), and the CT-FFR is expressed as a percentage. Pericoronary adipose tissue (PCAT) was defined as all voxels with CT attenuation ranging from −190 to −30 HU within a radial distance from the outer coronary wall equal to the average diameter of the respective coronary vessel. Automated software was utilized to segment the PCAT of target lesions on vessels after importing CCTA images into the CQK platform, and then pFAI values were obtained. If there were multiple lesions in one vessel, the adipose tissue around the highest stenosis lesion was segmented. For PCAT, when a patient had multiple vascular lesions, the pFAI values in each vessel were combined as the total pFAI. The largest measured value in each vessel was selected as the maximum pFAI. To reduce the error generated by automated segmentation of the images, the three-dimensional coronary artery segments and PCAT fragments were manually corrected by two experienced radiologists who were blinded to the clinical information.

### MRI acquisition

All brain MRI scans were carried out at a local hospital using a 3.0-tesla MRI scanner (Discovery MR 750, GE Healthcare). This scanner was equipped with an eight-channel head coil. Consistent MRI parameters were maintained during scans, including T1 FLAIR, T2-weighted imaging, diffusion-weighted imaging (DWI), and T2 FLAIR sequences. The T2 FLAIR sequence was used to observe WMH, applying parameters such as a repetition time/echo time/inversion time of 9,000 ms/120 ms/2,412 ms, a field of view of 256 × 256 mm, a matrix of 256 × 256, a flip angle of 160°, an echo chain of 18, a bandwidth of 50 kHz, a 5 mm section thickness, and a zero interslice gap. T1 FLAIR was used for white matter segmentation, with settings such as a 1,750 ms repetition time, a 24 ms echo time, a 256 × 256 mm field of view, a 256 × 256 resolution, a 111° flip angle, a 10 echo chain, a 31.25 kHz bandwidth, a 5 mm section thickness, and a zero interslice gap.

### Progression of longitudinal WMH volume

All T1 FLAIR and T2 FLAIR baseline images were imported into SPM12 software^1^ for registration, followed by automatic segmentation of the WMH using the lesion prediction algorithm (LPA) in the LST^2^ toolbox in SPM12, which was implemented in MATLAB (The MathWorks, Inc., Natick, United States). WMH volume was measured using the spatial dimensions of each MRI slice voxel (Schmidt et al., 2012). The process involved eliminating nonbrain matter and refining white matter segmentation. WMH volumes were calculated in milliliters, corrected for interscan intracranial volume differences, and normalized to the baseline intracranial volume. LPA does not require any parameters for user setting and is usually faster and more sensitive than the lesion growth algorithm (LGA). To minimize oversegmentation by LPA, the results were first viewed independently by two experienced radiologists. Then, the images segmented incorrectly by LPA were manually segmented and measured by an experienced neuroradiologist using itk-snap software.^3^ The change in WMH volume from the baseline to follow-up FLAIR images was documented. WMH volume fluctuation was classified into (1) progression and (2) nonprogression. A WMH change was considered progressive if the increase was more than 0.25 mL and there was significant visual change; if not, it was deemed nonprogressive. The smallest volume change is 0.25 mL, which is also likely be observed by radiologists. The benchmark was obtained from the 2015 study by Cho et al. (2015). Figures 2C–F shows the progression of WMH.

### Construction and assessment of the prediction model

The clinical details of patients with progressive WMH were divided into two categories in each dataset. We then compared the differences in CAC score between these categories to identify potential predictive variables. Univariate logistic regression was applied to each predictive factor in the training set. Next, three models were developed using multivariable logistic regression to assess WMH progression: Model 1 incorporated traditional cardiovascular risk factors; Model 2 included CT-FFR and pFAI on top of Model 1; and Model 3 was Model 2 plus the CAC index. Internal validation of the three models was performed with the validation set.

### Association between CAC score and WMH volume

WMH from each participant were segmented using LPA. Lesion probabilities were estimated for each voxel to segment WMH lesions observed in FLAIR images, and then local spatial lesion probability maps (LPMs) were calculated. The LPM was binarized (threshold = 0.5) with a minimum clustering range of 15 mm^3^ with default settings. To ensure accurate capture of WMH, all LPMs were visually inspected against the FLAIR images and manually corrected by an experienced radiologist according to published standards (Wardlaw et al., 2013b). We used the corresponding T1 images to calculate the binarized LPM for one participant in Mayo Clinic Adult Lifespan Template (MCALT) template space, which was then projected back to a single native space image using the inverse transform matrix calculated by the Advanced Neuroimaging Tool. All final templates warped to native space were visually inspected. Referring to MCALT,^4^ we extracted WMH volumes for nine regions: the deep white matter; corpus callosum; frontal, temporal, parietal, and occipital lobes; insula; brainstem; and cerebellum. The WMH volume of each brain region was normalized by dividing it by the intracranial volume. We performed a correlation analysis between the CAC scores of the 4 coronary arteries and the progression volumes of WMH in the 9 brain regions. Then, all the patients were divided into three groups according to CAC risk grade (Grade 1, *n* = 60; Grade 2, *n* = 43; Grade 3, *n* = 34). We further compared the WMH variations across the different brain regions between the three CAC risk groups.

### Statistical analysis

The statistical processing was done with SPSS 23.0 (IBM, Armonk, NY), MedCalc 19.3.1, and Excel 2019 (Microsoft). *p* < 0.05 was significant.

Categorical variables are presented as n (%), and parametric data are reported as mean ± SD, following the application of the Kolmogorov–Smirnov test for normality. The chi-square test or Fisher’s exact test was used for categorical variable comparisons, while Student’s t test was utilized for continuous variable comparisons. After a comparative analysis of the CAC score between the two datasets per risk factor, significant risk factors were incorporated into the univariate logistic regression to calculate their association with WMH progression. Three predictive models were formulated based on multivariable logistic stepwise regression in a training set to evaluate WMH progression. The models’ efficiency was validated using the receiver operating characteristic (ROC) curve, sensitivity, specificity, accuracy, precision, and F1-score. Finally, we compared the differences in WMH outcomes in different brain regions among patients in different CAC risk groups.

## Results

### Clinical characteristics

The study included 137 patients with WMH (mean age, 68.66 years ±9.64; 48 female), of whom 66 were put into the progression group (mean age, 70.42 years ±8.79; 24 female) and 71 patients (mean age, 67.01 years ±10.15; 24 female) were put into the nonprogression group. Statistical analysis revealed significant differences in age, hyperlipidemia, and alcohol abuse between the two groups (*p* < 0.05). No significant differences were observed in the other clinical factors, including sex, time interval, BMI, hypertension, diabetes mellitus, and smoking history (all *p* > 0.05). Further analysis showed significant differences in maximum pFAI and CT-FFR between the groups (*p* < 0.05), though total pFAI was similar (*p* > 0.05). The CAC scores of LM, LAD, LCX, and RCA and the total CAC score showed statistically significant differences (*p* < 0.05). The CAC risk grade also differed between the groups (*p* < 0.05). The findings are detailed in Table 1.

**Table 1:** Demographic, clinical, and imaging characteristics of patients with and without WMH progression.

Variable	All data (*n* = 137)	Progression of WMH		t or *x*^2^ or *z*	*p-*value
		Yes (*n* = 66)	No (*n* = 71)		
Demographics					
Female sex (n)	48 (35.0%)	24 (36.4%)	24 (33.8%)	0.099	0.754
Age (y)	68.66 ± 9.64	70.42 ± 8.79	67.01 ± 10.15	2.095	0.038*
Time interval (mo.)	24.11 ± 11.99	25.92 ± 11.94	22.41 ± 11.86	1.721	0.088
Cardiovascular risk factors					
BMI (kg/m^2^)	23.66 ± 3.77	23.76 ± 3.86	23.57 ± 3.71	0.298	0.766
Hypertension (n)	84 (61.31%)	46 (69.70%)	38 (53.52%)	3.773	0.052
Hyperlipidemia (n)	43 (31.39%)	28 (42.42%)	15 (21.13%)	7.204	0.007*
Diabetes mellitus (n)	52 (37.96%)	24 (36.36%)	28 (39.44%)	0.137	0.711
Smoking in past 5 years (n)	54 (39.42%)	28 (42.42%)	26 (36.62%)	0.483	0.487
Alcohol abuse in past 5 years (n)	37 (27.01%)	23 (34.85%)	14 (19.72%)	3.972	0.046*
pFAI and CT-FFR					
Total pFAI	−65.94 ± 21.69	−66.37 ± 7.09	−65.54 ± 29.44	−2.223	0.824
Maximum pFAI	−58.20 ± 12.94	−52.78 ± 15.04	−63.23 ± 7.88	5.037	0.000*
CT-FFR (%)	74.93 ± 5.82	72.67 ± 4.70	77.03 ± 6.00	−4.752	0.000*
CAC score					
LM	17.29 ± 55.28	26.18 ± 57.11	9.02 ± 52.58	−2.802	0.005*
LAD	146.03 ± 290.85	232.59 ± 381.88	65.56 ± 123.73	3.392	0.001*
LCX	41.32 ± 126.15	70.40 ± 173.76	14.29 ± 36.67	2.571	0.012*
RCA	8,202 ± 177.94	133.39 ± 223.88	34.27 ± 100.84	3.299	0.001*
Total CAC	286.66 ± 484.91	462.57 ± 625.01	123.13 ± 194.45	4.226	0.000*
CAC risk grade				29.763	0.000*
1 (n)	60 (43.80%)	12 (18.18%)	48 (67.61%)		
2 (n)	43 (31.39%)	28 (42.42%)	15 (21.13%)		
3 (n)	34 (24.82%)	26 (39.39%)	8 (11.26%)		

*Indicates statistical significance. WMH, white matter hyperintensity; CAC, coronary artery calcium. BMI, body mass index; pFAI, pericoronary fat attenuation index; CT-FFR, CT-fractional flow reserve; LM, left main artery; LAD, left anterior descending artery; LCX, left circumflex artery; RCA, right coronary artery.

### CAC score characteristics in the training and internal validation sets

A sample of 50 out of 137 cases was first randomly selected as the internal validation set, including 25 progression WMH and 25 nonprogression WMHs, and the remaining 87 cases were taken as the training set, which were 41 progressive and 46 nonprogressive cases (Figure 1 and Table 2). In the training set, there was no difference in the CAC score of LM between the WMH progression and nonprogression patients (*p* = 0.310), while other variables were significantly different (*p* < 0.05). In the internal validation set, all CAC scores were different between the WMH progressive and nonprogression groups (*p* < 0.05).

**Table 2:** Coronary artery calcium score of patients in the training and internal validation sets.

	Training set (*n* = 87)		*p-*value	Internal validation set (*n* = 50)		*p-*value
	Progression of WMH (*n* = 41)	No progression of WMH (*n* = 46)		Progression of WMH (*n* = 25)	No progression of WMH (*n* = 25)	
LM	26.51 ± 57.85	12.97 ± 65.10	0.310	25.64 ± 57.07	1.75 ± 5.70	0.048*
LAD	184.78 ± 171.46	70.81 ± 116.92	0.001*	311.00 ± 579.35	55.90 ± 137.37	0.041*
LCX	51.81 ± 83.26	16.79 ± 42.57	0.018*	100.89 ± 262.03	9.70 ± 22.12	0.096
RCA	155.21 ± 252.50	45.42 ± 123.30	0.014*	97.62 ± 165.37	13.75 ± 21.80	0.019*
Total CAC	418.31 ± 415.52	145.98 ± 211.12	0.000*	535.15 ± 872.58	81.10 ± 154.49	0.017*
CAC risk grade			0.000*			0.000*
1 (n)	7 (17.07%)	28 (60.9%)		5 (20.0%)	17 (68.0%)	
2 (n)	19 (46.34%)	12 (26.1%)		9 (36.0%)	6 (24.0%)	
3 (n)	15 (36.59%)	6 (13.0%)		11 (44.0%)	2 (8.0%)	

*Indicates statistical significance. WMH, white matter hyperintensity; CAC, coronary artery calcium. LM, left main artery; LAD, left anterior descending artery; LCX, left circumflex artery; RCA, right coronary artery.

### Factors associated with longitudinal WMH progression

**Figure 3:**
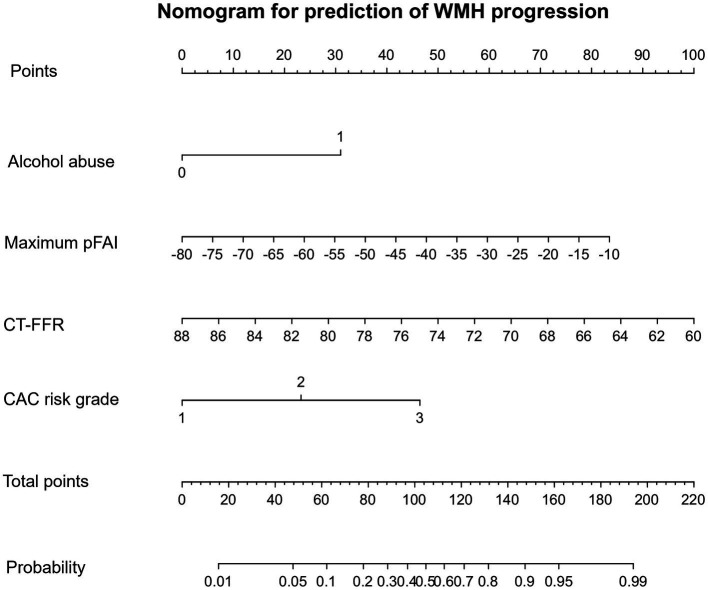
CAC nomogram for the prediction of WMH progression. The CAC nomogram was developed with alcohol abuse, maximum pFAI, CT-FFR, and CAC risk grade in the primary cohort. The nomogram displays the contribution of the four predictor variables to the probability of WMH progression in the future. Each variable is represented as a line on the graph, with its value mapped onto the y-axis. The values are all added to make the total score, which is then mapped onto the predicted probability scale to find the probability of WMH progression in the future.

A univariate logistic regression analysis revealed that factors such as hyperlipidemia, alcohol abuse, maximum pFAI; CT-FFR; CAC scores of LAD, LCX, and RCA; total CAC score; and CAC risk grade were associated with the progression of WMH. Multivariate logistic regression was utilized to develop the predictive model and establish a comprehensive nomogram (Table 3 and Figure 3). Longitudinally, the WMH volume increased in people who regularly engaged in alcohol abuse compared to those who did not [odds ratio (OR) = 5.262, 95% CI 1.397–19.820, *p* = 0.014]. The maximum pFAI (OR = 1.060, 95% CI 1.004–1.118, *p* = 0.036) was positively associated with progression, whereas the larger the CT-FFR was, the more likely WMH showed a nonprogressive trend (OR = 0.835, 95% CI 0.740–0.942, *p* = 0.003). The CAC risk grade was a strong predictor of WMH volume progression: With grade 1 as the reference, the ORs for grades 2 and 3 were 5.614 (95% CI 1.495–21.075; *p* = 0.011) and 9.985 (95% CI 2.197 ~ 45.389, *p* = 0.003), respectively (Table 3).

**Table 3:** Risk factors associated with the progression of WMH by univariate and multivariate logistic regression analysis.

Variable	Univariate logistic regression		Multivariate logistic regression	
	OR (95% CI)	*p-*value	OR (95% CI)	*p-*value
Demographics				
Sex (n)	0.933 (0.382 ~ 2.278)	0.879	NA	NA
Age (y)	1.040 (0.993 ~ 1.090)	0.094	NA	NA
Time interval (mo.)	0.998 (0.965 ~ 1.033)	0.919	NA	NA
Cardiovascular risk factors				
BMI (kg/m^2^)	1.005 (0.901 ~ 1.120)	0.930	NA	NA
Hypertension (n)	2.084 (0.805 ~ 5.397)	0.130	NA	NA
Hyperlipidemia (n)	3.511 (1.370 ~ 9.204)	0.009*	NA	NA
Diabetes mellitus (n)	0.566 (0.238 ~ 1.346)	0.198	NA	NA
Smoking in past 5 years (n)	1.974 (0.820 ~ 4.751)	0.129	NA	NA
Alcohol abuse in past 5 years (n)	3.566 (1.285 ~ 9.896)	0.015*	5.262 (1.397 ~ 19.820)	0.014
pFAI and CT-FFR				
Total pFAI	0.994 (0.974 ~ 1.014)	0.564	NA	NA
Maximum pFAI	1.073 (1.027 ~ 1.121)	0.002*	1.060 (1.004 ~ 1.118)	0.036*
CT-FFR (%)	0.840 (0.763 ~ 0.926)	0.000*	0.835 (0.740 ~ 0.942)	0.003*
CAC score				
LM	1.004 (0.996 ~ 1.012)	0.331	NA	NA
LAD	1.006 (1.002 ~ 1.010)	0.002*	NA	NA
LCX	1.011 (1.001 ~ 1.021)	0.032*	NA	NA
RCA	1.004 (1.000 ~ 1.008)	0.030*	NA	NA
Total CAC	1.003 (1.001 ~ 1.005)	0.001*	NA	NA
CAC risk grade				
1 (n)		0.000*		0.006*
2 (n)	6.333 (2.110 ~ 19.011)	0.001*	5.614 (1.495 ~ 21.075)	0.011*
3 (n)	10.000 (2.843 ~ 35.180)	0.000*	9.985 (2.197 ~ 45.389)	0.003*

*Indicates statistical significance. WMH, white matter hyperintensity; CAC, coronary artery calcium. BMI, body mass index; pFAI, pericoronary fat attenuation index; CT-FFR, CT-fractional flow reserve; LM, left main artery; LAD, left anterior descending artery; LCX, left circumflex artery; RCA, right coronary artery.

### Comparison of machine learning algorithms

All statistical analyses in the process of constructing the four models were performed using the Extreme Smart Analysis platform^5^ (Liu et al., 2022). The four machine learning algorithms were logistic regression, multilayer perceptron (MLP) classifier, support vector machine (SVM) and k-nearest neighbor (KNN). Detailed assessments of the predictive performance of the four machine learning models are provided in Table 4 and in Supplementary material. Our analysis showed that the logistic regression model outperformed the others in predicting WMH progression, with an area under the curve (AUC) of 0.864, sensitivity of 0.746, specificity of 0.866, and accuracy of 0.800. The recall rates of these models are shown in Supplementary Figure S1. We found that the logistic regression model yielded the highest AUC and recall values. We chose logistic regression as our final predictive model.

**Table 4:** Comparison of four machine learning algorithms.

Algorithms	Training set				Validation set			
	AUC	Sensitivity	Specificity	Accuracy	AUC	Sensitivity	Specificity	Accuracy
Logistic	0.864	0.746	0.866	0.800	0.814	0.690	0.883	0.686
MLP	0.422	0.196	0.919	0.561	0.473	0.343	0.914	0.571
SVM	0.758	0.559	0.889	0.721	0.761	0.575	0.931	0.700
KNN	0.858	0.748	0.778	0.736	0.731	0.667	0.732	0.657

AUC, area under the curve; MLP, multilayer perceptron classifier, SVM, support vector machine; KNN, k-nearest neighbor.

### Efficiency of different models for predicting the progression of WMH

**Figure 4:**
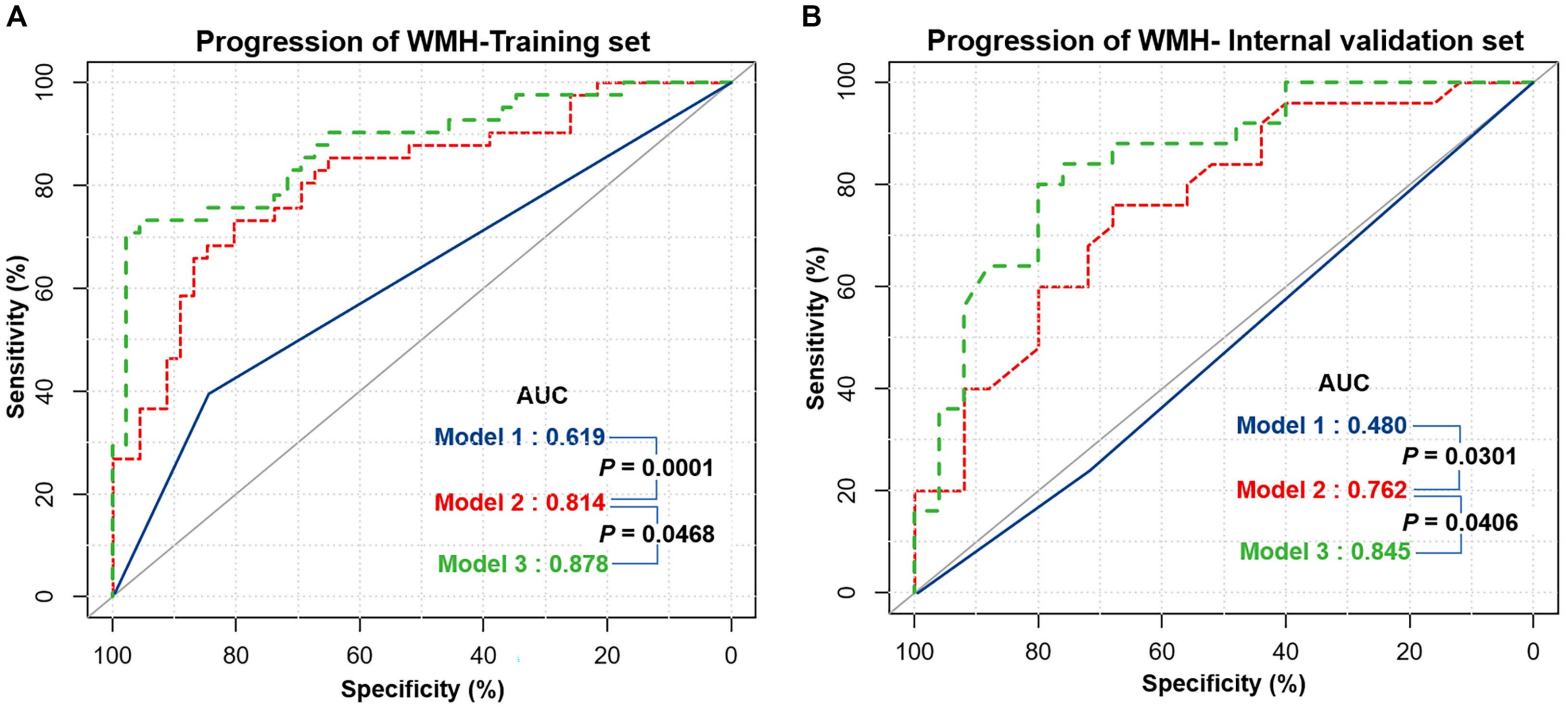
The receiver operating characteristic (ROC) curves of three different models for predicting the progression of white matter hyperintensity. **(A)** The performance of three models in the training set. **(B)** The performance of the three models in the internal validation set. The performance differences between the three models were compared with the DeLong test, and a *p-*value below 0.05 was considered significant. Model 1 = Alcohol abuse; Model 2 = Alcohol abuse plus maximum pFAI plus CT-FFR; Model 3 = Alcohol abuse plus maximum pFAI plus CT-FFR plus CAC risk grade. AUC, area under the curve.

**Figure 5:**
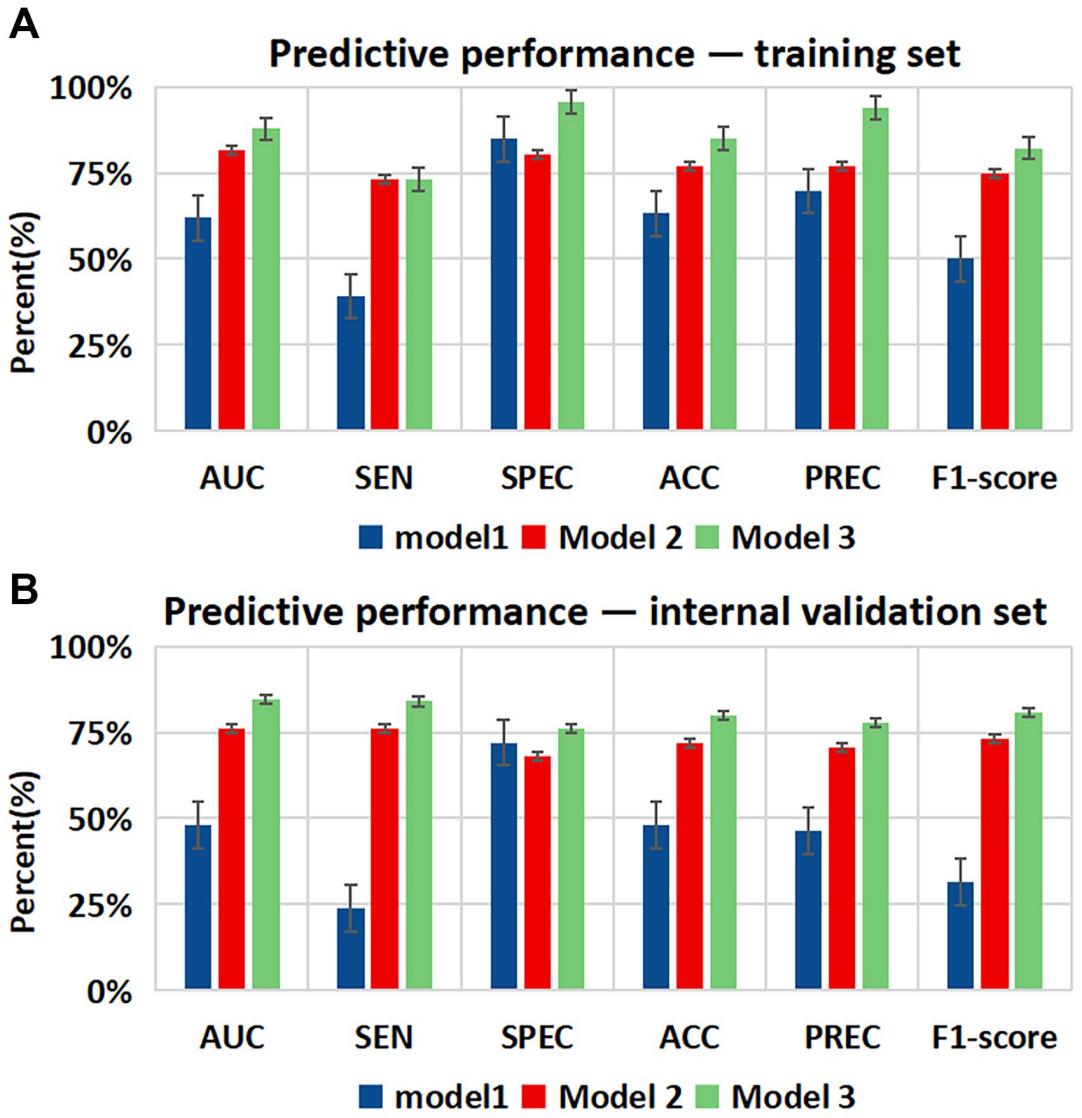
Bar charts showing diagnostic performance for identifying the progression of WMH in different datasets. Comparison of the area under the curve (AUC), sensitivity (SEN), specificity (SPE), accuracy (ACC), precision (PREC), and F1-score among the three models in the **(A)** training set and **(B)** internal validation set. Error bars indicate 95% CIs. Model 1 = Alcohol abuse; Model 2 = Alcohol abuse plus maximum pFAI plus CT-FFR; Model 3 = Alcohol abuse plus maximum pFAI plus CT-FFR plus CAC risk grade.

The progression of WMH was evaluated using three models developed from multivariable logistic regression analysis. Model 1 incorporated alcohol abuse, Model 2 included alcohol abuse and the maximum pFAI and CT-FFR, and Model 3 added the CAC risk grade. The predictive accuracies of these models are demonstrated in Figures 4, 5 and Table 5. Model 1 had a moderate AUC in the training set (AUC = 0.619, 95% CI: 0.500, 0.739) and in the internal validation set (AUC = 0.480, 95% CI: 0.319, 0.641). Including the quantitative CCTA-derived markers (maximum pFAI and CT-FFR) in Model 2 enhanced the AUC, achieving a better prediction of WMH progression (training set: AUC = 0.814, 95% CI: 0.723, 0.905; internal validation set: AUC = 0.762, 95% CI: 0.632, 0.895) than Model 1 (*p* < 0.05). The AUC further improved upon adding the CAC risk grade in Model 3, underscoring its enhanced predictive ability over Models 1 and 2 (training set: AUC = 0.878, 95% CI: 0.790, 0.938; internal validation set: AUC = 0.845, 95% CI: 0.734, 0.953).

**Table 5:** Comparison of efficiency in different models for predicting the progression of WMH.

	Training set (*n* = 87)			Internal validation set (*n* = 50)		
	Model 1	Model 2	Model 3	Model 1	Model 2	Model 3
AUC	0.619	0.814	0.878	0.480	0.762	0.845
Sensitivity	0.390	0.732	0.732	0.240	0.76	0.84
Specificity	0.848	0.804	0.956	0.720	0.68	0.76
Accuracy	63.2%	77.0%	85.1%	48.0%	72.0%	80.0%
Precision	69.6%	76.9%	93.8%	46.2%	70.4%	77.8%
F1 score	0.500	0.750	0.822	0.316	0.731	0.808

Model 1 = alcohol abuse; Model 2 = alcohol abuse plus maximum pFAI plus CT-FFR; Model 3 = alcohol abuse plus maximum pFAI plus CT-FFR plus CAC risk grade. WMH, white matter hyperintensity; AUC, area under the curve.

### Association between CAC score and WMH volumes

**Figure 6:**
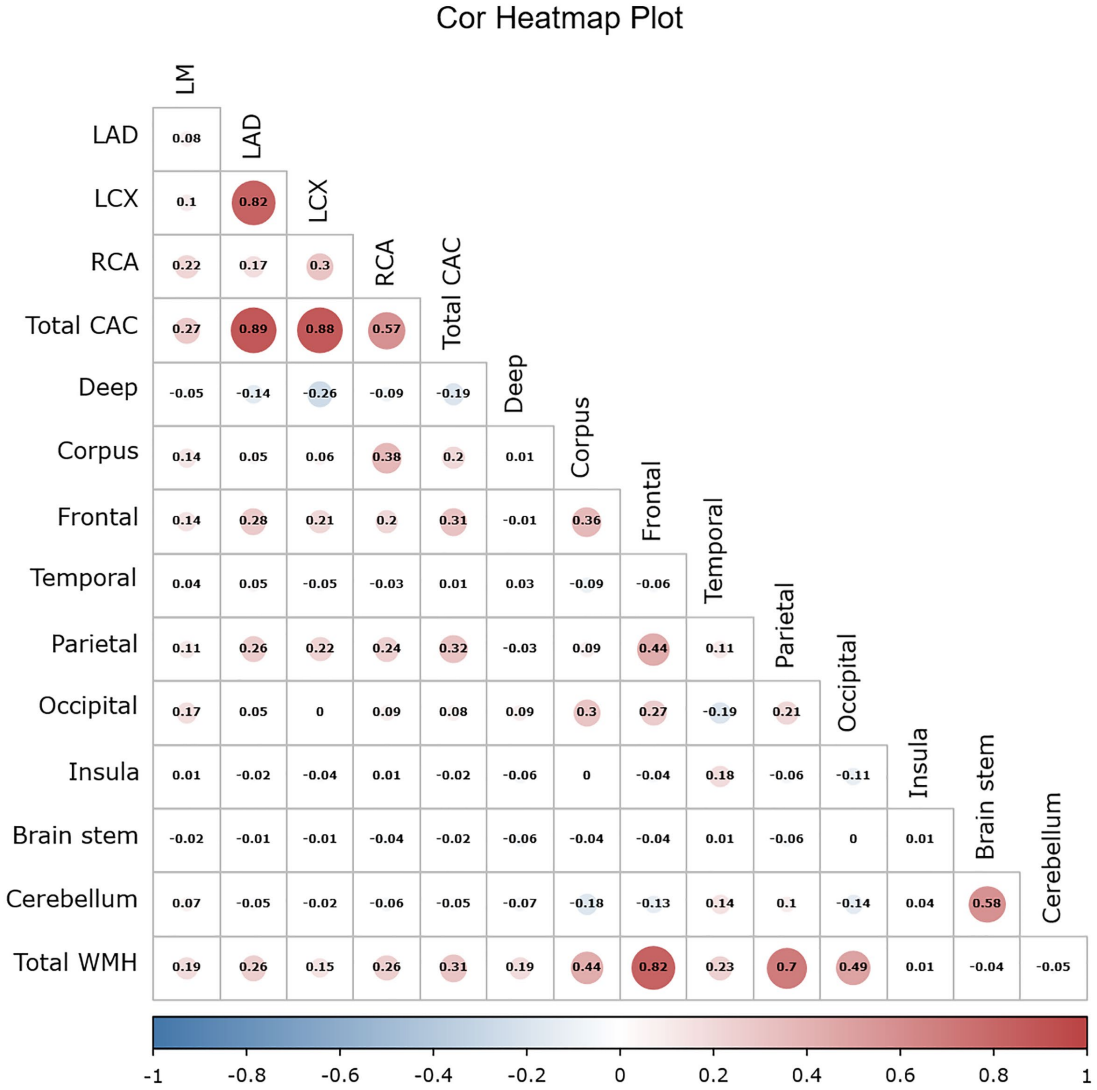
Simple correlation heatmap between the CAC score and the progression volume of WMH using the Pearson correlation coefficient. The CAC score included 4 coronary artery scores and the total score. The number indicates the value of the correlation coefficient, the bar indicates the range distribution of the correlation coefficient, and the color indicates the positive and negative of the correlation coefficient as well as the magnitude, with darker color indicating higher correlation. WMH volumes included the volumes in the nine regions of the brain and the total volume.

**Figure 7:**
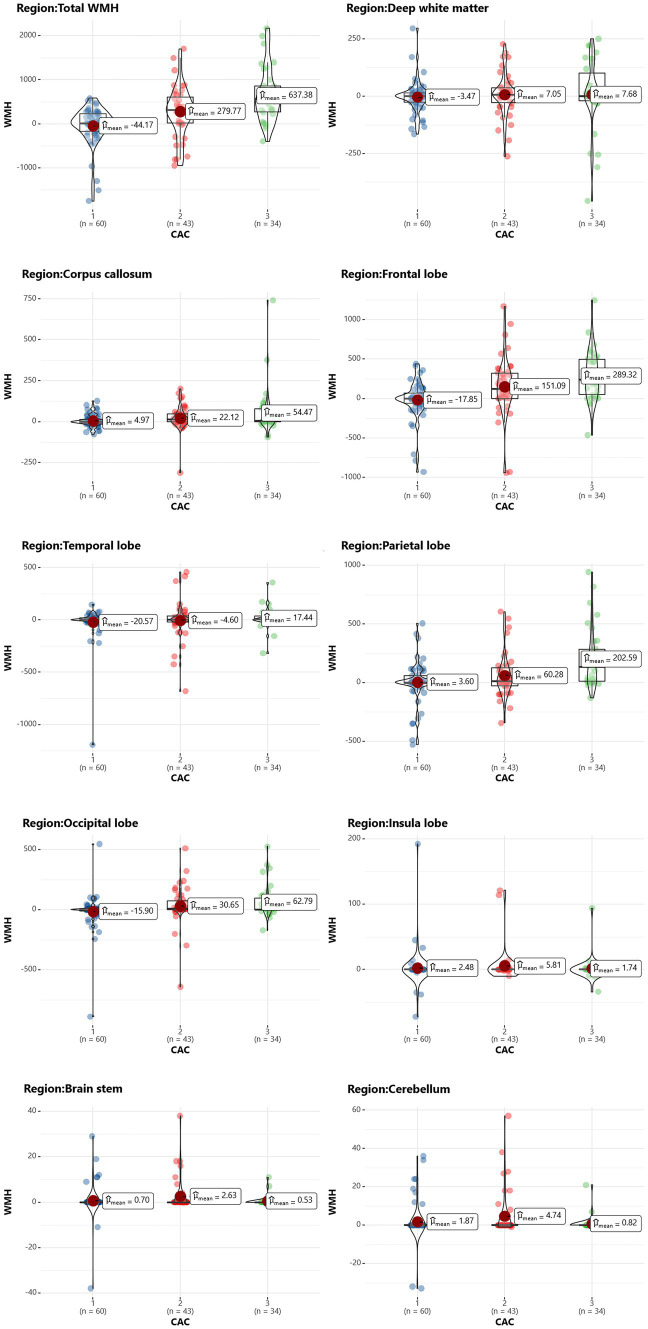
Scatterplot of the WMH change in each brain region in different CAC risk groups. The vertical axis represents the volume change in WMH (in mL), and the horizontal axis represents the CAC risk grade.

Using the Pearson correlation coefficient to evaluate the quantitative correlation between CAC score and WMH progression volume, we found that there was no significant correlation between any individual CAC score and WMH progression volume in any individual brain region (*r* < 0.4) (Figure 6). Significant differences were noted in the total WMH volume change between the three CAC risk grade groups (*p* < 0.05), greater changes becoming evident as the CAC risk grade increased (Grade 1: −44.17 ± 438.48 mL; Grade 2: 279.77 ± 572.95 mL; Grade 3: 637.38 ± 607.34 mL). Links were observed between CAC risk grade and the WMH volume changes in the corpus callosum (*p* = 0.045), frontal lobe (*p* < 0.05), parietal lobe (*p* < 0.05), and occipital lobe (*p* = 0.047). No link was found between the WMH volume change and CAC risk grade in the deep white matter, temporal lobe, insula, brain stem or cerebellum (*p* > 0.05). More specific observations are detailed in Table 6 and Figure 7.

**Table 6:** The difference between CAC risk grade and the progression volume of WMH in different regions.

WMH region	CAC score			F or χ^2^	*p-*value	*Post hoc*
	Grade 1 (*n* = 60)	Grade 2 (*n* = 43)	Grade 3 (*n* = 34)			
Total WMH	−44.17 ± 438.48	279.77 ± 572.95	637.38 ± 607.34	18.42^a^	<0.05*	i, ii, iii
Deep white matter	−3.47 ± 68.15	7.05 ± 94.00	7.68 ± 154.02	2.53^b^	0.282	/
Corpus callosum	4.97 ± 37.14	22.12 ± 76.05	54.47 ± 146.45	6.21^b^	0.045*	i, ii
Frontal lobe	−17.85 ± 237.98	151.09 ± 381.83	289.32 ± 317.13	26.01^b^	<0.05*	i, ii
Temporal lobe	−20.57 ± 163.63	−4.60 ± 187.20	17.44 ± 99.92	0.62^a^	0.537	/
Parietal lobe	3.60 ± 173.77	60.28 ± 185.22	202.59 ± 256.03	15.47^b^	<0.05*	ii, iii
Occipital lobe	−15.90 ± 148.07	30.65 ± 164.00	62.79 ± 140.78	3.32^a^	0.047*	ii
Insula lobe	2.48 ± 3.67	5.81 ± 3.84	1.74 ± 3.03	0.31^a^	0.732	/
Brain stem	0.70 ± 7.37	2.63 ± 7.32	0.53 ± 2.21	2.55^b^	0.279	/
Cerebellum	1.87 ± 10.43	4.74 ± 12.02	0.82 ± 3.76	1.80^b^	0.407	/

^a^Means ANOVA. ^b^Means Kruskal–Wallis test. *p* < 0.05. *Indicates a significant difference between groups. *Post hoc* analysis: i: Grade 1 vs. Grade 2; ii: Grade 1 vs. Grade 3; iii: Grade 2 vs. Grade 3. WMH, white matter hyperintensity; CAC, coronary artery calcium.

## Discussion

Identifying reliable biomarkers for predicting WMH progression is clinically significant, as it enables early identification and intervention in high-risk patients. The present study evaluated the utility of the CAC score as a predictor of the progression of WMH in patients with CAD. We conducted both univariate and multivariate logistic regression analyses to identify independent risk factors for WMH progression and developed three predictive models from them. We compared the predictive performance of traditional clinical risk factors, CCTA-derived markers (CT-FFR and pFAI) and CAC markers. Finally, we partitioned the regions of WMH progression to compare the progression of WMH volumes in different brain regions between different CAC risk grade subgroups. Our study had three major findings: (1) Alcohol abuse, maximum pFAI, CT-FFR and CAC risk grade were independent predictors of WMH progression (*p* < 0.05). (2) The combined model with alcohol abuse, maximum pFAI, CT-FFR and CAC risk grade showed the best performance in predicting the progression of WMH (training set: AUC = 0.878, 95% CI: 0.790, 0.938; internal validation set: AUC = 0.845, 95% CI: 0.734, 0.953). (3) As the CAC risk grade increased, the volume change in the total WMH (*p* < 0.05), corpus callosum (*p* = 0.045), frontal lobe (*p* < 0.05), parietal lobe (*p* < 0.05), and occipital lobe (*p* = 0.047) became more significant.

WMH, a ubiquitous characteristic of the aging brain, is typically associated with various neurological disorders. CAC is a noninvasive method of assessing the risk of cardiovascular disease. Past studies hint at potential shared pathways linking CAC and WMH, including atherosclerosis and arterial endothelial barrier damage (Wardlaw et al., 2003, 2017). Our previous study also found an association between CAC score and WMH volume, and a high-risk CAC score had a greater effect on WMH volume in the elderly population. In this study, we employed an automated segmentation method to measure WMH volume, offering a more objective, accurate, and consistent approach than the Fazekas visual scale used in earlier studies. Our study discovered that the CAC risk grade independently influences the likelihood of WMH progression. We observed a pronounced increase in the risk of progression with a higher CAC risk grade. This indicates that the cumulative effect of CAC, an atherosclerosis marker, could be linked to cognitive decline and dementia risk (Reis et al., 2013; Kuller et al., 2016; Fujiyoshi et al., 2017). Intriguingly, several cardiovascular risk aspects, such as hypertension, hyperlipidemia, and diabetes, did not predict WMH progression in our study, challenging earlier research findings. This discrepancy may be due to interference from other factors during the multivariate regression analysis. In a prospective study among older adults, hypertension strongly predicted progressive white matter hyperintensity (Maillard et al., 2012; Choi et al., 2022). These discrepancies made us consider whether long-lasting antihypertensive treatment could decelerate WMH progression, thus decreasing its predictive power. Furthermore, our findings counter the classically held belief about age as a determinant of WMH progression, though this is in alignment with findings that revealed declining vascular density as an age-independent pathological mechanism (Moody et al., 2004).

In this study, before establishing the prediction model for WMH progression, we first resampled the complete data, implementing a fivefold cross-validation. We evaluated four common machine learning algorithms, finding that logistic regression performed best in the training and validation sets. We constructed the predictive model by logistic regression. Traditional cardiovascular risk factors showed limited predictive efficacy for WMH progression with a training set AUC of 0.619 and a validation set AUC of 0.480. Upon incorporating CCTA-derived biomarkers, the AUC significantly improved, with a training set AUC of 0.814 and a validation set AUC of 0.762. The most effective model, Model 3, featured the CAC risk grade, improving the performance to a training set AUC of 0.878 and a validation set AUC of 0.845. Recent research has pointed out that CAC is an indicative marker of atherosclerosis and is closely tied to cardiovascular risk factors. The findings study signify that CAC, compared to myocardial-derived markers derived from CCTA, is more effective at predicting the progression of WMH. This discovery shows that the degree of CAC can be intuitively assessed based on CCTA to determine the impact of cardiovascular diseases on WMH.

A previous study used radiomics features of white matter throughout the brain, including histogram features, form factor features, co-occurrence matrix (GLCM) features, and run-length matrix (RLM) features, to predict the progression of any, periventricular, and deep WMH. The area under the curve (AUC) values for the training and testing datasets were 0.697–0.758 (Shu et al., 2020). Our results show that the CAC risk grade was better at predicting the progression of total WMH. We further partitioned the whole-brain WMH into 9 regions, comparing the correlation between CAC and volume changes of WMH in different brain regions. The results suggest that CAC affects different brain functions, enhancing our understanding of the heart-brain correlation. Our analysis showed that there was no significant correlation between the CAC score of the four coronary arteries and the WMH volume in various brain regions. However, between the different CAC risk subgroups, WMH progression volumes were significantly different in the corpus callosum and frontal, parietal and occipital lobes. These four regions are extremely important parts of the brain that are involved in processing our memory, thinking, sensation, and vision. WMH could have negative effects on these functions, such as a decline in memory and impaired cognitive function. Therefore, predicting and assessing WMH based on the CAC score is important to prevent the onset of neurodegenerative diseases.

Explaining the clinical significance of research results is an important aspect of scientific studies. As a retrospective study, this cross-sectional design cannot definitively draw causal links between CAC scores and WMH progression. However, these preliminary statistical results reflect the correlation between CAC scores and WMH progression. In the future, it is necessary to explore the expected variations in the progression of WMH across different CAC risk tiers through prospective design. Understanding these variations can help improve risk assessment and guide treatment decisions. In practical settings, utilizing automated software for CAC scoring can reduce the subjectivity of manual assessments. Combining machine learning models for automated prediction of WMH progression can provide medical professionals with valuable tools. Medical professionals can categorize patients into different risk categories based on CAC scores, allowing for more informed decisions regarding treatment strategies. For instance, patients with higher CAC scores indicate a significant plaque burden and may require more aggressive interventions such as lipid-lowering medications or invasive procedures. Meanwhile, incorporating CAC scores into early screening strategies can help identify high-risk individuals with cerebral small vessel disease. Furthermore, investigating whether the progression of WMH at different risk grades is associated with specific clinical outcomes, such as cognitive decline or the occurrence of related cardiovascular events, can guide personalized treatment plans and improve patient prognosis.

This study has several limitations. First, the patients were elderly adults with CAD, and they appeared to have more vascular risk factors, so the applicability of the findings to younger demographics or those without CAD may be limited. And the sample size of 137 patients was relatively small, potentially affecting the generalizability. Furthermore, the WMH volume changed excessively in some patients, which could affect the comparison of results between patients. However, the results demonstrated the potential for the CAC score to predict the progression of WMH, and in the future, we will include more individuals to validate the accuracy of these findings. Second, the lack of publicly available datasets related to our aims lowers its credibility. Unfortunately, we were unable to access relevant data for this purpose during the study. Our data suggested that the time interval between the two MRI scans did not significantly impact the progression of WMH, possibly due to the small sample size and short time intervals. The progression value of WMH in this study was an absolute increment, without considering the influence of the baseline size of WMH, and the measurement errors of the quantitative values may have potential effects on the experimental results, which makes it necessary to further improve the precision of WMH segmentation. Finally, as with all traditional machine learning methods, our developed model may be enhanced further if radiomics or deep learning features are incorporated. To improve the model’s robustness and generalizability, we plan to extract radiomics features of plaques and leverage deep learning algorithms to train the data.

In conclusion, the CCTA-derived CAC score, along with specific risk factors, can predict the progression of WMH in patients with coronary artery disease. In the future, we may be able to identify people at increased risk for catastrophic vascular events by deploying a CAC score to achieve early detection of and intervention against WMH.

## Data availability statement

The datasets presented in this article are not readily available because legal restrictions, imposed by the ethical committee, prevent us from publicly sharing the dataset due to sensitive patient information. Requests to access the datasets should be directed to HJ, 2857865081@qq.com; XG, gong.xy@vip.163.com.

## Ethics statement

The studies involving humans were approved by the Ethics Committee of ZJPP Hospital. The studies were conducted in accordance with the local legislation and institutional requirements. Written informed consent for participation was not required from the participants or the participants’ legal guardians/next of kin in accordance with the national legislation and institutional requirements.

## Author contributions

HJ: Conceptualization, Data curation, Formal analysis, Investigation, Methodology, Writing – original draft, Writing – review & editing. JH: Conceptualization, Data curation, Formal analysis, Investigation, Methodology, Writing – original draft, Writing – review & editing. XQ: Data curation, Formal analysis, Investigation, Writing – review & editing. XD: Data curation, Investigation, Writing – review & editing. GZ: Formal analysis, Investigation, Writing – review & editing. YM: Data curation, Formal analysis, Writing – review & editing. ZS: Software, Writing – review & editing. YW: Formal analysis, Software, Resources, Writing – review & editing. XG: Conceptualization, Data curation, Formal analysis, Funding acquisition, Investigation, Supervision, Writing – review & editing.

## Footnotes

^1^https://www.fil.ion.ion.ucl.ac.uk/spm/software/spm12/

^2^https://www.statistical-modelling.de/lst.html

^3^http://www.itksnap.org/pmwiki/pmwiki.php

^4^https://www.nitrc.org/projects/mcalt/

^5^https://www.xsmartanalysis.com/
