# Supplementary material for: Predicting progression of white matter hyperintensity using coronary artery calcium score based on coronary CT angiography—feasibility and accuracy
Source: Front Aging Neurosci. 2023 Nov 10;15:1256228. doi: 10.3389/fnagi.2023.1256228 (PMC10667909; doi:10.3389/fnagi.2023.1256228)
Supplement: Supplementary file 1 [file Data_Sheet_1.docx]

**Supplementary Material**

**Predicting Progression of White Matter Hyperintensity Using Coronary Artery Calcium Score Based on Coronary CT Angiography - Feasibility and Accuracy**

Hui Jin^1,2^ †, Jie Hou^1^ †, Xue Qin^2^, Xingyue Du^2^, Guangying Zheng^1^, Yu Meng^1^, Zhenyu Shu^1^, Yuguo Wei^3^, Xiangyang Gong^1*^

***Correspondence:** Xiangyang Gong: gong.xy@vip.163.com

**1. Comparison of machine learning algorithms**

Four machine learning algorithms were used to try to complete the classification task of all data samples, including logistic regression, multilayer perceptron (MLP) classifier, support vector machine (SVM) and k-nearest neighbor (KNN). The parameter values of each model are selected as follows:

a. Logistic Regression:

C (regularization factor): 1.0

max_iter (maximum number of iterations): 100

penalty (type of regularization): L2

tol (convergence tolerance): 0.0001

b. MLP Classifier:

Activation function (non-linear function): ReLU

Hidden layer sizes: (20, 10)

Maximum number of iterations: 20.

c. SVC:

C (regularization factor): 1.0

Kernel type: RBF

Tolerance for stopping criterion: 0.001.

d. K-Nearest Neighbor:

Number of neighbors: 5

Weight function used in prediction: uniform.


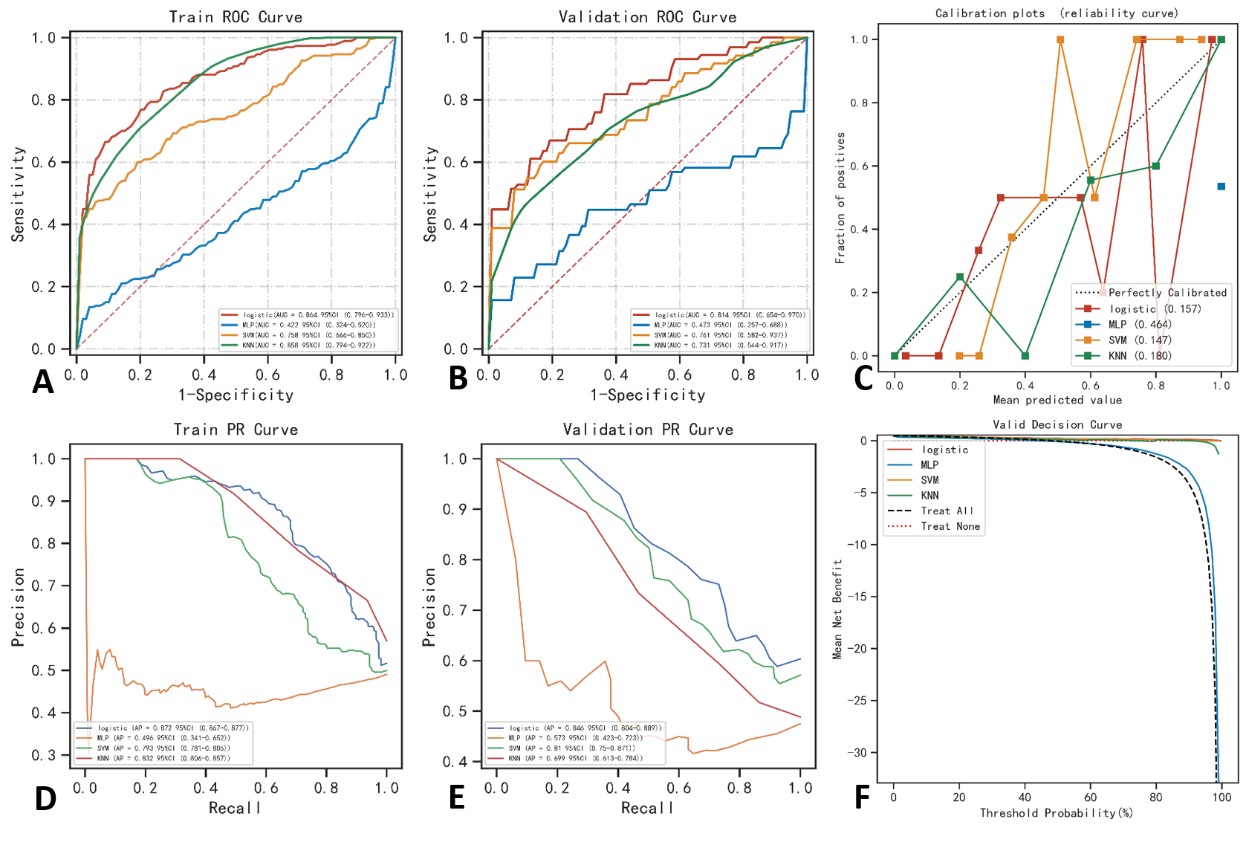


**Supplementary Figure S1.** Performance of four machine learning algorithms for predicting the progression of WMH

**2.** **The process of score increment analysis**

Logistic regression was used to score the variable set, with the following model parameters:

C (regularization factor): 1.0

max_iter (maximum number of iterations): 100

penalty (type of regularization): L2

tol (convergence tolerance): 0.0001

The process of score increment analysis is as follows:

(1) Determine the importance ranking of the variable set [Alcohol abuse, Maximum pFAI, CT-FFR and the CAC risk grade] through prior estimation.

(2) Take alcohol abuse as the initial model, and then add [Maximum pFAI, CT-FFR and the CAC risk grade] into the model one by one and compare the performance indicators of the models containing 2, 3 and 4 variables.

(3) Compare the performance of models containing different numbers of variables to determine the best variable set. According to the results, the best variable set is [Alcohol abuse, Maximum pFAI, CT-FFR and the CAC risk grade].


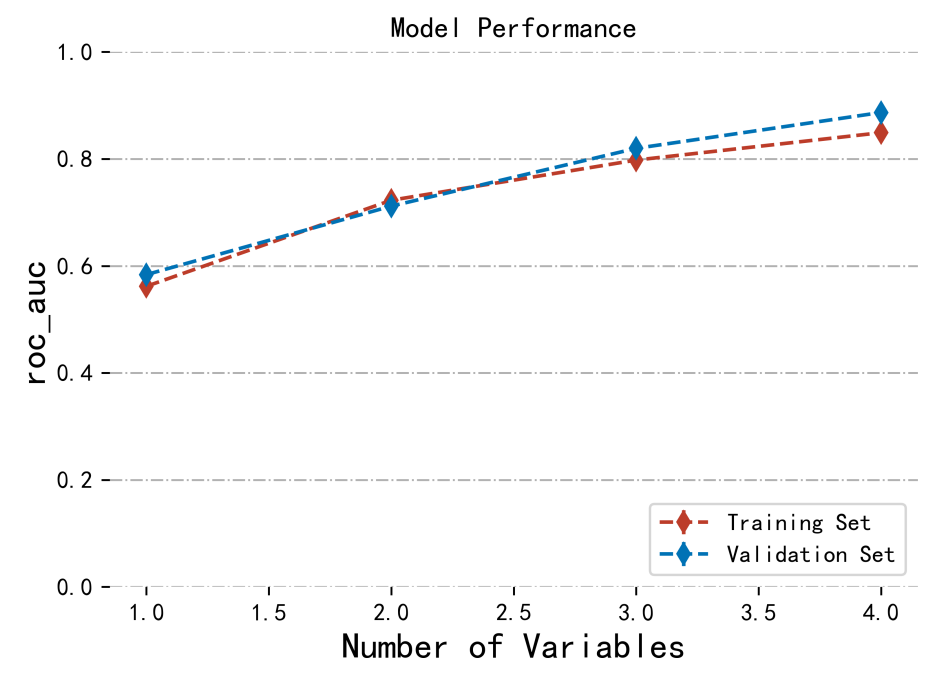


**Supplementary Figure S2.** The score increment using logistic regression in training and validation sets
